# Peripheral nerve injury increases glutamate-evoked calcium mobilization in adult spinal cord neurons

**DOI:** 10.1186/1744-8069-8-56

**Published:** 2012-07-28

**Authors:** Suzanne Doolen, Camille B Blake, Bret N Smith, Bradley K Taylor

**Affiliations:** 1 Department of Physiology, School of Medicine, University of Kentucky Medical Center, Lexington, KY, 40536, USA

**Keywords:** Pain, AMPA receptor, Central sensitization, Calcium imaging

## Abstract

**Background:**

Central sensitization in the spinal cord requires glutamate receptor activation and intracellular Ca^2+^ mobilization. We used Fura-2 AM bulk loading of mouse slices together with wide-field Ca^2+^ imaging to measure glutamate-evoked increases in extracellular Ca^2+^ to test the hypotheses that: 1. Exogenous application of glutamate causes Ca^2+^ mobilization in a preponderance of dorsal horn neurons within spinal cord slices taken from adult mice; 2. Glutamate-evoked Ca^2+^ mobilization is associated with spontaneous and/or evoked action potentials; 3. Glutamate acts at glutamate receptor subtypes to evoked Ca^2+^ transients; and 4. The magnitude of glutamate-evoked Ca^2+^ responses increases in the setting of peripheral neuropathic pain.

**Results:**

Bath-applied glutamate robustly increased [Ca^2+^]_i_ in 14.4 ± 2.6 cells per dorsal horn within a 440 x 330 um field-of-view, with an average time-to-peak of 27 s and decay of 112 s. Repeated application produced sequential responses of similar magnitude, indicating the absence of sensitization, desensitization or tachyphylaxis. Ca^2+^ transients were glutamate concentration-dependent with a K_d_ = 0.64 mM. Ca^2+^ responses predominantly occurred on neurons since: 1) Over 95% of glutamate-responsive cells did not label with the astrocyte marker, SR-101; 2) 62% of fura-2 AM loaded cells exhibited spontaneous action potentials; 3) 75% of cells that responded to locally-applied glutamate with a rise in [Ca^2+^]_i_ also showed a significant increase in AP frequency upon a subsequent glutamate exposure; 4) In experiments using simultaneous on-cell recordings and Ca^2+^ imaging, glutamate elicited a Ca^2+^ response and an increase in AP frequency. AMPA/kainate (CNQX)- and AMPA (GYKI 52466)-selective receptor antagonists significantly attenuated glutamate-evoked increases in [Ca^2+^]_i_, while NMDA (AP-5), kainate (UBP-301) and class I mGluRs (AIDA) did not. Compared to sham controls, peripheral nerve injury significantly decreased mechanical paw withdrawal threshold and increased glutamate-evoked Ca^2+^ signals.

**Conclusions:**

Bulk-loading fura-2 AM into spinal cord slices is a successful means for determining glutamate-evoked Ca^2+^ mobilization in naïve adult dorsal horn neurons. AMPA receptors mediate the majority of these responses. Peripheral neuropathic injury potentiates Ca^2+^ signaling in dorsal horn.

## Background

Central sensitization is a glutamate receptor-dependent increase in the excitability of neurons within the central nervous system, and is thought to contribute to enhanced responsiveness to sensory input following tissue injury [1]. After nerve injury, damaged and non-damaged peripheral afferents generate spontaneous action potentials (AP) within nociresponsive neurons within the dorsal horn [2]. Glutamate, released from pre-synaptic terminals in an activity-dependent manner, binds post-synaptic ionotropic and metabotropic receptors, leading to intracellular Ca^2+^ mobilization [1,3,4]. The resulting calcium-induced activation of intracellular kinases can then phosphorylate several residues on the C-terminus of NMDA and AMPA receptors; these post-translational modifications, together with increased receptor trafficking to and from the membrane and increased responsiveness to glutamate, are thought to contribute to the functional changes that manifest as central sensitization [5-7]. Despite the importance of Ca^2+^ mobilization to the central sensitization that drive neuropathic pain, however, few studies have investigated real-time [Ca^2+^_i_ responses in populations of dorsal horn neurons [8,9].

While patch-clamp recording allows sampling of single neurons, the bulk-loading of Ca^2+^-sensitive indicator dyes enables simultaneous recording across populations of dorsal horn cells. However, previous studies describing bulk-loaded Ca^2+^ imaging in spinal cord slices have not fully discriminated between neuronal and glial responses [8-10]. One obstacle is that both neurons and astrocytes exhibit glutamate-evoked Ca^2+^mobilization in the CNS [11,12]. To overcome this obstacle in our spinal cord slice imaging studies, we employed the fluorescent dye SR-101, a specific marker of astroglia in the CNS [13,14].

Behavioral pharmacology studies support the contribution of spinal glutamatergic and Ca^2+^ channel signaling to the development and/or maintenance of allodynia and hyperalgesia [15-18]. For example, it is well-known that intrathecal AMPA- and NMDA-receptor antagonist administration attenuates pain-like behavior in models of neuropathic pain [15-19]. Furthermore, convergent observations obtained in a variety of pain models indicate that intrathecal administration of antagonists to N- and T-type voltage-gated calcium channels (VGCC) produce anti-allodynic effects, with smaller effects after injection of P/Q- and L-type channel blockers [20-26]. While these studies implicate glutamate receptors and Ca^2+^ signaling in central sensitization and neuropathic pain, the underlying cellular mechanisms are poorly understood. In this study, we show that glutamate-evoked Ca^2+^ signals in adult dorsal horn neurons are mediated predominantly by AMPA channels and are potentiated by peripheral neuropathic injury.

## Results

### Glutamate-evoked Ca^2+^ mobilization in adult dorsal horn neurons

**Figure 1:**
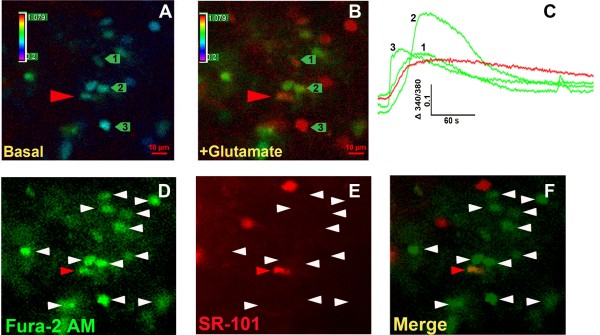
** Calcium imaging in adult mouse spinal cord slices.** Ratiometric images of dorsal horn before ( **A**) and during ( **B**) a 10 s exposure to 1 mM glutamate. Pseudocolor images show values of the 340/380 ratio where blue indicates basal Ca^2+^ levels and green/red indicate a glutamate-evoked Ca^2+^ increase. **C**. Glutamate-evoked Ca^2+^ traces evoked in panels A and B. The red trace represents an SR-101 positive cell indicated by the red arrowhead in A and B and the green traces represent SR-101 negative cells indicated by green arrows. Numbers on green arrows correspond to numbered traces in panel **C**. **D**. Numerous cells bulk loaded with Fura-2 AM are indicated by white arrowheads. A double-labeled astrocytic cell is indicated by a red arrowhead. **E**. Astrocyte labeling pattern with SR-101 in the same adult spinal cord slice. **F**. Merged image shows that the majority (~95%) of Fura-2 AM loaded cells are non-astrocytic.

To determine whether exogenous application of glutamate causes Ca^2+^ mobilization in a preponderance of lamina II dorsal horn cells of adult mice, we conducted Fura-2 Ca^2+^ imaging within transverse spinal cord slices. Spontaneous calcium waves were not observed, consistent with previous reports in 11–15 day-old mice [10]. Within a 440 x 330 um field-of-view in 9 dorsal horn slices, we observed an average of 15 ± 1 Fura-2 loaded cells. Imaged cells were located less than 35 um below the tissue surface. Glutamate superfusion (1 mM, 10 s) evoked Ca^2+^ transients in 93% (14 ± 2) cells (Figure 1 and Table 1). Glutamate increased [Ca^2+^_i_ with an average time-to-peak and duration (from onset to return to 20% of peak) of approximately 27 s and 139 s, respectively.

**Table 1:** **SR-101 labeling and action potentials in cells that respond to glutamate with an increase in [Ca**^**2+**^**]**_**i**_

**# cells exhibiting glutamate-evoked increase [Ca**^**2+**^**]**_**i**_	**SR-101 positive cells**	**Double labeled cells**	**% Double labeled cells**
**130 (14 ± 2.6)**	**149 (16 ± 1.8)**	**6 (1 ±0.23)**	**4.36 ± 1.4**
**# cells exhibiting glutamate-evoked increase [Ca**^**2+**^**]**_**i**_	**# cells exhibiting Spontaneous AP**		**%**
**29**	**18**		**62.1**
	**# cells exhibiting glutamate-evoked AP**		**%**
**20**	**15**		**75**

Data represent total cell counts. Average ± SEM per slice shown in parenthesis are from 9 slices from 9 different animals.

Both CNS neurons and astrocytes respond to glutamate with intracellular Ca^2+^ mobilization [11,12]. To discriminate the two cell types, we used real-time double-labeling with fura-2 and SR-101. Previous studies indicate that incubation of brain slices with SR-101 specifically labels astrocytes for at least 8 h [13,14]. As illustrated in Figure 1 and Table 1, SR-101 labeled 16 ± 1.8 cells within a 440 x 330 um field-of-view in the dorsal horn (n = 9 views). Of the 130 glutamate-responsive cells, 4 ± 1.4% (6 cells) were labeled with SR-101. Of the SR-101 positive cells, only a small proportion, 4 ± 1.7%, were glutamate responsive. These data suggest that the vast majority of glutamate-responsive cells are SR-101 negative, and thus likely represent non-astrocytic cells.

**Figure 2:**
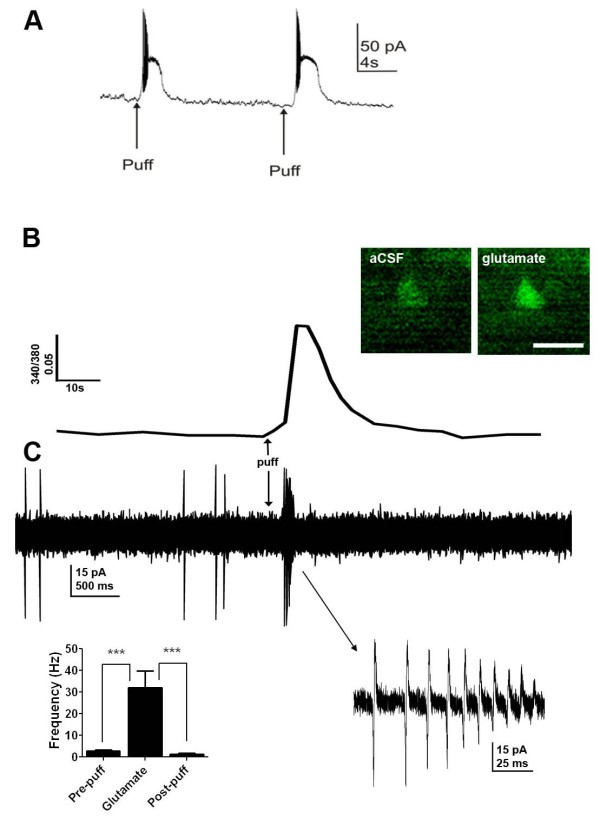
** Glutamate-evoked Ca**^**2+**^**response and APs in DH neurons.A**. Electrophysiological responses to glutamate are also repeatable. Whole cell recording from slices prepared from a post-natal day 13 mouse shows current responses to glutamate (50 mM; 200 ms). **B**. Ca^2+^ response to glutamate (50 mM; 50 ms pulse). Inset: Fluorescent images taken with excitation at 340 nm before (left) and during exposure to glutamate (right). Scale bar: 10 um. **C**. Simultaneous on-cell recording indicates glutamate-evoked AP currents concurrently with Ca^2+^ response. Inset: Left: AP frequency over time. Right: Enlarged view showing generation of AP currents. *** P < 0.0001.

To further test the hypothesis that glutamate-evoked Ca^2+^ responses in spinal cord slices occur primarily in neurons, we conducted experiments to concurrently assess Ca^2+^ mobilization and electrophysiological responses to glutamate. We evaluated whole-cell recordings in 29 fura-2 loaded cells. Spontaneous APs were observed in 18 neurons (62.1%), with an average firing frequency of 2.97 ± 0.63 Hz. We found 20 cells that responded to a brief pulse of glutamate with a [Ca^2+^]_i_ increase (at least 10% above baseline). Of these, 15 (75%) exhibited a significant increase in AP frequency upon a subsequent glutamate exposure (from 3.0 ± 6 Hz in ACSF to 29.0 ± 8.7 Hz after glutamate, p < 0.01, n = 15; paired *t*-test). Figure 2A shows that glutamate caused a transient increase in AP firing.

**Figure 3:**
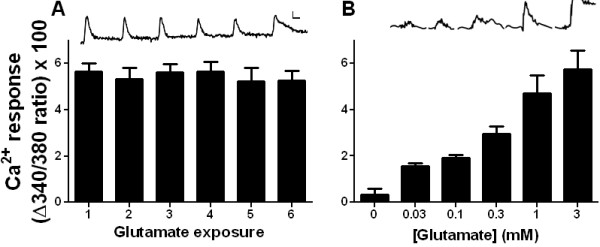
** Calcium responses elicited by glutamate.** ( **A**.- **B**.) Average peak Ca^2+^ responses are repeatable and concentration dependent. Slices prepared from 4–6 wk CD-1 mice were superfused with aCSF at 1 ml/min. Scale bar: horizontal, Δ340/380 = 0.1; vertical, time = 60 s. Data represent average ± SEM. **A.** Representative tracings and average peak Ca^2+^ responses to 10-s pulses of 1 mM glutamate spaced at 10 min intervals. Glutamate elicited repeatable [Ca^2+^]_i_ transients. N = 4 slices. **B.** Representative tracings and average peak Ca^2+^ responses to increasing glutamate concentrations. N = 3 slices.

On-cell recording allows for simultaneous electrophysiological recording and Ca^2+^ imaging because it employs a GΩ seal without rupturing the cell and allowing intracellular fura-2 to diffuse into the recording pipette. As illustrated in Figure 2B, experiments using simultaneous on-cell recordings and Ca^2+^ imaging, Ca^2+^ responses to glutamate were accompanied an increase in AP frequency. For both whole-cell and on-cell recordings, glutamate-evoked AP burst after focal glutamate application onset was rapid (2.54 ± 1.0 s), corresponding temporally with the Ca^2+^ rise. Action potential bursts (1.02 ± 0.38 s; n = 15) were typically of shorter duration than the corresponding Ca^2+^ response (34.03 ± 10.26 s) (Figure 3). To further characterize [Ca^2+^]_i_ responses, we administered glutamate repeatedly and at varying concentrations. As illustrated in Figure 3A, repeated superfusion with glutamate (1 mM for 10 s at 10 min intervals) produced sequential responses of similar magnitude (F_(5,18)_ = 0.43, p = 0.82), indicating the absence of sensitization, desensitization or tachyphylaxis.

### Glutamate receptor pharmacology

AMPAR, NMDAR and kainate receptor (KAR), as well as metabotropic (mGluRs, groups I–III) glutamate receptors are densely expressed in the dorsal horn and contribute to the initiation and maintenance of central sensitization [1,3,27-37].To confirm that glutamate acted through a receptor-dependent mechanism, we first evaluated [Ca^2+^_i_ responses to increasing glutamate concentrations. As illustrated in Figure 3B, the magnitude of glutamate-evoked increases in [Ca^2+^_i_ was concentration–dependent, with an ED_50_ = 0.64 mM.

**Figure 4:**
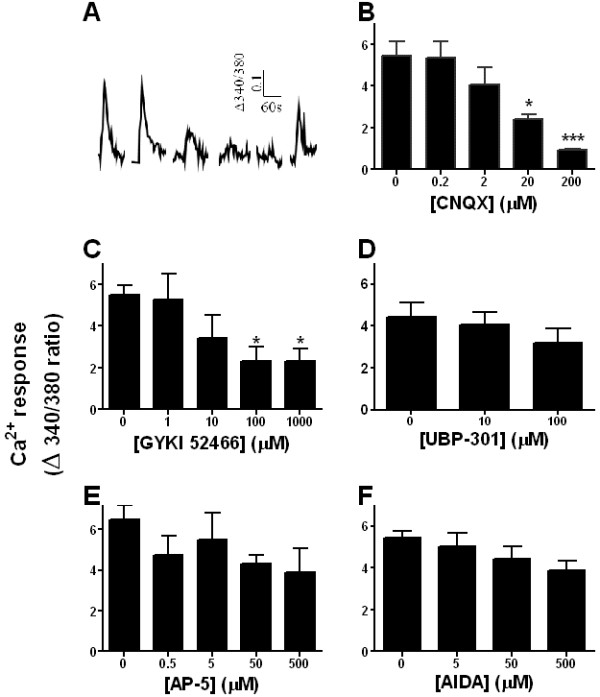
** Effect of sub-type selective antagonists on glutamate-evoked Ca**^**2+**^**responses.** Slices prepared from 4–6 wk CD-1 mice were superfused with aCSF at 1 ml/min. **A**. Representative Ca^2+^ traces from dorsal horn cell in response to 1 mM glutamate in the presence of increasing concentrations of CNQX. The last trace is after a final washout, indicating that the tissue is still viable and responsive to glutamate. **B**, CNQX (3–6 slices); **C**, GYKI 52466 (4–14 slices); **D**, UBP-301 (3 slices); **E**, AP-5 (4–11 slices); **F,** AIDA, (3–8 slices). Antagonists were present for 10 min prior, as well as during glutamate-evoked stimulation. Data represent average ± SEM. *P < 0.05.

To investigate which ionotropic receptor subtype(s) contribute to glutamate-evoked Ca^2+^ responses, spinal cord slices were pre-incubated with selective receptor antagonists. The repeatability of glutamate responses allowed for a simple, within-subject design involving glutamate application followed by antagonist administration prior to and during a second glutamate application. Ca^2+^ transients in response to glutamate in the presence of increasing concentrations of the AMPA/kainate receptor antagonist CNQX are shown in Figure 4A. CNQX attenuated glutamate-evoked Ca^2+^ transients in a concentration-dependent manner with an IC_50_ = 6.5 μM. The highest concentration tested (200 μM) produced an 83.1 ± 2.9% reduction (Figure 4B; F_(4,13)_ = 8.33, p < 0.01). Similarly, GYKI 52466, a selective AMPA receptor antagonists, attenuated glutamate-evoked Ca^2+^ transients with an IC_50_ = 6.1 μM. The maximal effect of GYKI 52466 observed at 100 μM was 56.7% (Figure 4C; F_(4,30)_ = 4.25, p < 0.01). By contrast, the kainate-selective antagonist UBP-301 did not significantly attenuate glutamate-evoked Ca^2+^ responses (Figure 4D; F_(2, 18)_ = 0.89, p = 0.42). Neither did UBP-310, another selective kainate antagonist (p > 0.05, data not shown). AP-5, an NMDA-selective antagonist, also did not change glutamate-evoked Ca^2+^ responses (Figure 4E; F_(4, 26)_ = 1.78, p = 0.34).

In addition to evoking Ca^2+^ influx through ionotropic glutamate receptors, glutamate can activate group I metabotropic receptors. Group I mGluRs couple to G_q_/_11_[38] and mobilize Ca^2+^ release from intracellular stores while group II and III couple to G_i/o_. Although group I receptors do not participate in basal nociception, their activation is necessary for activity-dependent central sensitization [39-44]. As illustrated in Figure 4F, however, the group I metabotropic glutamate receptor antagonist AIDA (up to 500 μM) did not significantly change Ca^2+^ responses as compared to vehicle (F_(3,15)_ = 2.14, p = 0.13).

### Glutamate-evoked Ca^2+^ mobilization in neuropathic injury

**Figure 5:**
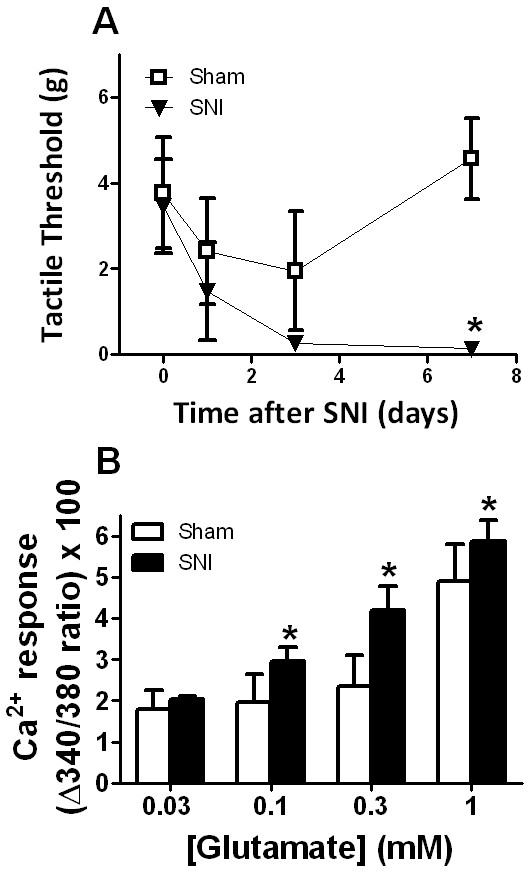
** Effect on peripheral nerve injury on glutamate-evoked Ca**^**2+**^**transients.A**. Development of mechanical hyperalgesia following SNI. Withdrawal threshold of the ipsilateral hindpaws to mechanical stimulation with von Frey hairs in SNI mice are compared to those of sham-operated mice. **B**. Peak glutamate-evoked Ca^2+^ responses in dorsal horn slices from sham and neuropathic (SNI) mice. Slices were prepared from 5 wk mice 7–10 d after sham or SNI surgery. Ca^2+^ responses were evoked by a 10 s exposure to glutamate at concentrations indicated. Data represent average ± SEM from 5 slices. *P < 0.05.

Behavioral pharmacology studies support the contribution of glutamatergic and Ca^2+^ channel signaling to the development and/or maintenance of neuropathic pain [15-19]. To test the hypothesis that peripheral nerve injury potentiates glutamate-evoked Ca^2+^ responses in a manner that coincides with neuropathic pain-like behavior, we first evaluated tactile sensitivity prior to, 1d, 3d and 7d following Sham or SNI surgery. Figure 5A illustrates the development of mechanical hyperalgesia at 7 days post-SNI surgery. The force required to elicit hindpaw withdrawal decreased from a pre-surgical mean baseline value of 3.5 ± 1.1 to a much lower value on day 7 after SNI of 0.1 ± 0.04. Thus, compared to sham controls, SNI significantly reduced hindpaw withdrawal threshold (F_(2,40)_ =4.66, p < 0.05). We next evaluated the glutamate dose-response relationship of peak Ca^2+^ signals in slices obtained 7–10 days after surgery, (Figure 5B). Compared to Sham, SNI produced significantly greater glutamate-evoked Ca^2+^ signals (F_(1,11)_ = 9.8, p < 0.05). Bonferroni post-hoc analysis found this difference to be significant at all concentrations above 0.03 mM.

## Discussion

The present study characterizes glutamate-evoked Ca^2+^ mobilization in adult dorsal horn and its modulation by neuropathic injury. For the first time, we report simultaneous Ca^2+^ imaging of multiple neurons in adult mouse spinal cord slices using real-time imaging of Fura-2 and SR 101. Our results reflect an assay in which glutamate predominantly evokes Ca^2+^ signals in neurons, and indicated that this is largely mediated by AMPA receptors. Our results indicate that SNI potentiates Ca^2+^ signaling and may lead to spinal cord central sensitization and neuropathic pain.

### Glutamate superfusion evokes repeatable, concentration-dependent Ca^2+^ mobilization in the dorsal horn of adult spinal cord slices

*In vitro* spinal cord slice studies of pain transmission most commonly use tissues obtained from neonatal rodents [10,45,46]. Since AMPA subunit mRNA levels [47] and both NMDA and AMPA receptor subunit protein expression in the spinal cord are high during development and decline until 4 weeks of age [48] plasticity in response to neuropathic or inflammatory insult is not clear. We chose to study the mechanisms responsible for Ca^2+^ signaling in adults because glutamate signaling in rodents is not mature until 4 wk of age [47-53]. It is also important to characterize Ca^2+^ mobilization in adult spinal cord since many animals models of pain take many weeks to establish.

A previous Ca^2+^ imaging study in transverse slices from adult spinal cord indicated that imaging was unsatisfactory in slices from animals older than 15 days [10,54], perhaps due to poor fura-2 uptake into the tissue. However, with the use of ice cold transcardial perfusion with aCSF containing kynurinic acid, rapid spinal cord isolation, and a slicer with minimal advance speed and minimal blade deflection, we were able to elicit glutamate-evoked [Ca^2+^_i_ increases in nearly all fura-2 loaded cells from young adult animals. Furthermore, the magnitude of [Ca^2+^_i_ responses was concentration-dependent, and sustained with repeated applications of glutamate. These results indicate superb tissue health of our slice preparation, and demonstrate access of the cells to the superfusion fluid.

Glutamate concomitantly evoked APs and robust increases in [Ca^2+^_i_. The Ca^2+^ response peaked at approximately 30 sec and typically lasted 2–3 minutes, consistent with the temporal profile of [Ca^2+^_i_ increase following NMDA superfusion in Fura-2-injected single cells of 18–30 d old spinal cord slices [55]. By contrast, the duration of neuronal firing during the glutamate puff application lasted about 1 second. This most likely resulted from inactivation of Na^+^-dependent APs caused by the continued and strong depolarization of the neurons by glutamate. Our whole-cell voltage tracings suggest that the glutamate-dependent depolarization persisted even after sodium channels were inactivated. This sustained depolarization may reflect or contribute to the substantial Ca^2+^ mobilization following glutamate stimulation, which takes several minutes to return to basal [Ca^2+^_i_ levels. Further experiments will be necessary to determine the relative contribution of APs and VGCC opening, Ca^2+^ entry through glutamate receptors, or intracellular Ca^2+^ release from stores to the glutamate-evoked [Ca^2+^_i_ rise.

### Glutamate-evoked Ca^2+^ mobilization occurs predominantly in neurons

Sandkuhler and colleagues were the first to describe Ca^2+^ responses in adult transverse spinal cord slices pre-loaded with fura-2 [8]. Both neurons and astrocytes exhibit glutamate-evoked calcium mobilization in the CNS [11,12,56]. Sandkuhler and colleagues defined a Ca^2+^ response to be neuronal if it peaked within 1 s of stimulus initiation, and its amplitude exceeded ten times baseline noise [8]. They also stated that patch-clamp recordings from fura-2-loaded dorsal horn cells suggested responses to be neuronal; however, they did not present data confirming this finding [8]. Because more recent studies indicate that mechanical sensory stimulation can elicit Ca^2+^ transient peaks in astrocytes within 0.5 s [57], we sought more direct evidence indicating that Ca^2+^ responses to glutamate occur predominantly in neurons. First, we found that less than 5% of glutamate-responsive cells were labeled with the astrocyte marker, SR-101. This is consistent with recent *in vivo* studies from the hindlimb area of the mouse primary somatosensory cortex, which demonstrated that ~5% of SR-101 labeled astrocytes exhibit Ca^2+^ transients in response to mechanical hindlimb stimulation [57]. On the other hand, ~80% of SR101 cells were stained with fluo-4 AM in juvenile hippocampal slices from rats [58]. Different cell types in CNS tissue slices acquire Ca^2+^-sensitive dyes with different efficiency, presumably as a result of variability in cellular surface-to-volume ratio [59]. We speculate that, in our spinal cord slice preparation, fura-2 is particularly facile at crossing the neuronal membrane.

A second piece of evidence indicating that Ca^2+^ responses predominantly occur on neurons is our electrophysiological data, which suggests that the majority of fura-2 loaded cells are excitable. For example, 62% of fura-2 loaded cells exhibited spontaneous APs and 75% of cells that responded to glutamate with a rise in [Ca^2+^]_i_ also showed a significant increase in AP frequency upon a subsequent glutamate exposure. In experiments using simultaneous AP recordings and Ca^2+^ imaging, glutamate elicited a Ca^2+^ response and an increase in AP frequency. We conclude that, since the majority of glutamate-responsive fura-2 loaded cells are non-astrocytic and are electrically excitable, neurons make up the majority of responsive cells.

### Glutamate-evoked Ca^2+^ mobilization is predominantly driven by AMPA receptor activation

The AMPA/kainite and AMPA receptor-selective antagonists CNQX and GYKI 52466 substantially inhibited glutamate-evoked increases in [Ca^2+^_i_, while kainate-, NMDA-, and metabotropic class I- selective antagonists did not. These data suggest that in dorsal horn of adult mice, AMPA receptors are sufficient to drive glutamate-evoked Ca^2+^ signaling, either directly or by eliciting APs, which activate VGCCs. This is not surprising because at resting conditions the NMDA receptor channel pore is blocked by Mg^2+^[60]. This contrasts with results obtained in spinal cord slices from juvenile mice where both NMDA and AMPA receptors contribute to Ca^2+^ mobilization in response to dorsal root stimulation [10]. One explanation for these disparate findings is that, relative to neonate, adult NMDA receptors play a relatively small role in glutamate-evoked Ca^2+^ mobilization. More specifically, NR2B mRNA is present in lamina II during embryonic development through adulthood [61]. The NR2B subunit is characterized by a relatively strong Mg^2+^ block [52], and therefore AMPA contribution may not be sufficient to relieve the Mg^2+^ block from NMDA receptors in adult slices. Conversely, the NR2D subunits that are highly expressed in dorsal horn at P1 and P7 (but absent by P14) [61], are characterized by a relatively weak Mg^2+^ block [52], and therefore AMPA contribution would be sufficient to relieve the Mg^2+^ block from NMDA receptors in juvenile slices. A second possible explanation is that the dorsal root stimulation used in the juvenile slice studies provides synaptically localized glutamate release that is sufficient to activate NMDA receptors, whereas our concentrations of superfused glutamate do not reach sufficiently high concentrations to elicit NMDA receptor activation. Indeed, we found that the AP-5 produced a trend towards inhibition of glutamate-evoked Ca^2+^ response, which is similar to the small 20% decrease observed in the setting of dorsal root stimulation [55].

Possible mechanisms by which the AMPA receptor contributes to glutamate-evoked Ca^2+^ mobilization include depolarization-induced expulsion of the NMDA Mg^2+^ block, thus allowing Ca^2+^ entry through the NMDA receptor [62]. Alternatively, Ca^2+^ may enter the pore of calcium-permeable AMPA receptors. Agonist-induced cobalt-uptake, a surrogate marker for agonist-induced calcium influx, has revealed a high density of Ca^2+^-permeable AMPA receptors in the spinal dorsal horn, particularly in superficial laminae [63]. Activation of AMPA receptors can also induce APs. Since glutamate most often resulted in AP generation in dorsal horn neurons, the depolarization, consequent opening of VGCCs, and subsequent Ca^2+^ flux is a likely contributor to the Ca^2+^ mobilization we observed.

Our findings that class I mGlu receptors do not contribute to Ca^2+^ mobilization is in agreement with previous studies [10]. Previous studies also support our finding that kainate receptors do not contribute to Ca^2+^ mobilization: although kainate receptors on DRG neurons are predominantly Ca^2+^-permeable in late embryonic and newborn rats, they become fully Ca^2+^-impermeable later in the first postnatal week [34]. While these data suggest that kainate and class I mGlu receptors do not contribute to Ca^2+^ mobilization in naïve animals, they do not rule out the possibility that they contribute to central sensitization after nerve injury [39-44,64].

### Neuropathic injury potentiates glutamate-evoked Ca^2+^ mobilization

Our studies demonstrate that nerve injury produced mechanical hypersensitivity and potentiated glutamate-evoked Ca^2+^ mobilization in adult dorsal horn neurons. These data are consistent with behavioral pharmacology studies supporting the contribution of glutamatergic and Ca^2+^ channel signaling to the development and/or maintenance of neuropathic pain [15-18]. By what mechanism does SNI potentiate glutamate evoked increases in [Ca^2+^_i_? We present several possibilities. 1) One possible mechanism is that injury-induced afferent activity might drive the entry of Ca^2+^ through NMDA channels. After peripheral nerve injury, damaged and uninjured A- and C-fibers begin to generate spontaneous APs, leading to the release of primary afferent neurotransmitters [2]. Slow depolarization induced by peptides such as substance P may then lead to expulsion of Mg^2+^ block, allowing for NMDA channel opening and Ca^2+^ entry upon glutamate stimulation [65]. 2) A second possible explanation is that injury-induced activation of kinases may lead to the phosphorylation and subsequent alteration of NMDA and/or AMPA receptors. For example, upon noxious stimulation, PKA phosphorylates GluR1 subunits, causing insertion of these receptors into the post-synaptic membrane, thus leading to an increase in synaptic strength [31,66,67]. Also, noxious stimuli cause phosphorylation of NMDA NR1 subunits by PKA or PKC, leading to enhanced response to glutamate. [68-71]. 3) A third mechanism is that SNI increases mGluR activation of G_q/11_ thus leading to increased Ca^2+^ mobilization. Peripheral inflammation causes trafficking of group I mGluRs to the plasma membrane in the spinal dorsal horn neurons [36] and behavioral studies indicates a greater response to group I mGluR agonists in CCI compared to control, suggesting that group I mGluRs contribute to neuropathic pain [72]. 4) A fourth possible mechanism is that SNI induced a switch from GluR2-containing to GluR2-lacking AMPARs; this would allow for increased Ca^2+^ entry through the AMPA receptor pore. Ca^2+^ permeability is modified not only by neuronal activity, but also by changes in expression, trafficking post-translation changes of AMPAR subunits [73,74]. Studies using inflammatory pain models indicate activity-dependant GluR1 expression on the cell membrane and GluR2 internalization [75,76]. 5) A fifth possible mechanism is that astrocytic Ca^2+^ elevations induce NMDA receptor-mediated slow inward currents (SICs) and Ca^2+^ elevations in neurons, leading to pain sensitization. Experimentally induced stimuli that trigger Ca^2+^ mobilization in astrocytes (low Ca^2+^, BzATP) induce NMDA receptor-mediated SICs in neurons in the rat dorsal horn, and these SICs are significantly increased by inflammatory injury [77]. Our finding that glutamate induces Ca^2+^ elevations in a subset of astrocytes support the presence of this mechanism in dorsal horn. 6) A sixth, more general mechanism is that injury increases the electrophysiological properties of the post-synaptic neurons. While chronic sciatic constriction in rat did not change resting membrane potential, response threshold, or input resistance, it did increase the amplitude and frequency of subthreshold synaptic currents in a subset of lamina II neurons [78]. Ultimately, one or more likely several of these mechanisms could increase neuronal Ca^2+^ mobilization, a critical step to central sensitization.

## Methods

### Animals

Adult male CD-1 mice (4–8 weeks old) were housed 4 per cage in a temperature controlled (68-72  F) room on a 14:10 hour light/dark cycle (dark hours from 8 PM-6 AM), and were given food and water *ad libitum*. Animal use protocols were approved by the Institutional Animal Care and Use Committee of the University of Kentucky.

### Preparation of adult mouse spinal cord slices

Mice were anesthetized with 5% isoflurane and quickly perfused transcardially with 10 ml of ice-cold sucrose-containing artificial cerebrospinal fluid (aCSF) (sucrose-aCSF) that contained (in mM): NaCl 95, KCl 1.8, KH_2_PO_4_ 1.2, CaCl_2_ 0.5, MgSO_4_ 7, NaHCO_3_ 26, glucose 15, sucrose 50 kynurenic acid 1, oxygenated with 95% O_2_, 5% CO_2_; pH 7.4. The lumbar spinal cord was rapidly (within 90s) isolated by laminectomy from the cervical enlargement to the cauda equina, placed in oxygenated ice-cold sucrose-aCSF, cleaned of dura mater and ventral roots, and super-glued vertically to a block of 4% agar (Fisher Scientific, Pittsburgh, PA) on the stage of a Campden 5000mz vibratome (Lafayette, IN). Transverse slices (300–450 um) from lumbar segments L4-L5 were cut in ice-cold sucrose-aCSF using minimum forward speed ranging from 0.03 to 1 mm/s and using maximum vibration. The ideal total dissection and slicing time to insure slice viability was 22 minutes or less.

### Fluorometric Ca^2+^ measurements

Slices were incubated for 60 min at room temperature with Fura-2 AM (10 μM), pluronic acid (0.1%) in oxygenated aCSF containing (in mM): NaCl 127, KCl 1.8, KH_2_PO_4_ 1.2, CaCl_2_ 2.4, MgSO_4_ 1.3, NaHCO_3_ 26, glucose 15, followed by a 20 min de-esterification period in normal aCSF. Prior to recording, slices were kept at RT in a chamber containing approximately 150 ml of oxygenated aCSF. In some studies, prior to incubation in fura-2, slices were incubated for 20 min at 34  C in 1 μM SR-101 in normal aCSF, as previously described [14].

Slices were perfused at 1–2 ml/min with normal aCSF in an RC-25 recording chamber (Warner Instruments, Hamden, CT) mounted on a Nikon FN-1 upright microscope fitted with a 79000 ET FURA2 Hybrid filter set (Nikon Instruments, Melville, NY) and a Photometrics CoolSNAP HQ_2_ camera (Tucson, AZ). Relative intracellular Ca^2+^ levels were determined by measuring the change in ratio of fluorescence emission at 510 nm in response to excitation at 340 and 380 nm (200 ms exposure). Paired images were collected at 1–1.5 seconds/frame. Relative changes in Ca^2+^ levels were evaluated using Nikon Elements software by creating a region of interest over the cell body and calculating the peak change in ratio. The peak magnitude of Ca^2+^ transient was expressed as difference in (ratio x 100) exposure to exogenous glutamate compared to baseline before glutamate. The criteria for a Ca^2+^ response were considered at least 10% increase above the baseline ratio. Ca^2+^ transients were in response to a 10 s exposure to 1 mM glutamate unless indicated otherwise. In antagonist studies, the slice was perfused with the antagonist for 10 min prior to and during glutamate stimulation. Only cells that displayed a consistent control response to 1 mM glutamate at the beginning and end of the experiment (showing a less than 40% decrease in glutamate-evoked Ca^2+^ transients) of were included in this study.

### Electrophysiology

Cell-attached and/or whole-cell current-clamp recordings were obtained from dorsal horn neurons of the spinal cord under visual guidance on an Olympus BX51WI upright, fixed stage microscope equipped with infrared differential interference contrast (IR-DIC) and fluorescence optics (Olympus, Pittsburgh, PA). The aCSF used for simultaneous Ca^2+^ imaging and electrophysiological recording consisted of (in mM): 124 NaCl, 3 KCl, 2 CaCl_2_, 1.3 MgCl_2_, 1.4 NaH_2_PO_4_, 26 NaHCO_3_, and 11 glucose (pH 7.15–7.3); osmolality 290–310 mOsm/kg. Recording pipettes with open resistance of 2–5 MΩ were pulled from borosilicate glass capillaries with 0.45 mm wall thickness (King precision Glass, Claremont, CA). Seal resistance was 1–5 GΩ and, for whole-cell recordings, series resistance was 5–25 MΩ, uncompensated. Patch pipettes were filled with (in mM): 130 K^+^-gluconate, 1 NaCl, 5 EGTA, 1 MgCl_2_, 1 CaCl_2_, 3 KOH, 4 ATP; pH = 7.2-7.4. Neural activity was recorded using a Multiclamp 700B patch-clamp amplifier (Axon Instruments, Sunnyvale, CA), low-pass filtered at 5 kHz, acquired and with a Digidata 1440A digitizer and pClamp 10.3 software. Action potentials and whole-cell Na^+^ currents were analyzed off-line on a PC-style computer with pCLAMP programs (Axon Instruments) or Minianalysis 6.0.3 (Synaptosoft, Fort Lee, NJ). For whole-cell recordings, APs were recorded at rest in current-clamp mode. A 2-fold increase in firing frequency was used as minimum threshold for responses to glutamate stimulation.

For electrophysiology studies, chemical stimulation of neurons in the dorsal horn was made by pressure applying L-glutamate (50 mM; 10–200 ms; dissolved in aCSF) through a glass pipette (∼10 μm tip diameter) positioned at the surface of the slice (Picospritzer, Parker-Hannefin, Fairfield, NJ, USA). Glutamate was applied directly over the recoded cell to evoke unclamped, rapid APs and/or Ca^2+^ response. Slices were positioned such that aCSF flowed dorsolaterally, away from the dorsal horn to minimize possible direct effects of glutamate on other neurons in the slice during recording.

### Spared nerve injury (SNI) model

Mice were anesthetized with isoflurane (5% induction, 3% maintenance). The left hind-leg area was shaved and wiped clean with alcohol and an incision was made in the skin at the level of the trifurcation of the sciatic nerve. The overlying muscles were retracted, exposing the common peroneal, tibial, and sural nerves. Common peroneal and tibial nerves were transected. Care was taken to avoid touching or stretching the sural branch. The muscle was sutured with absorbable 6–0 sutures (Ethicon, Somerville, NJ) and the wound was closed with 9-mm metal clips. Sham surgery was produced by skin incision at the level of the trifurcation, and by exposing but not touching the sciatic branches.

### Behavioral tests

Tactile threshold was assessed with an incremental series of eight von Frey filaments of logarithmic stiffness (Stoelting, Inc., Wood Dale, IL; approximately 0.008–6.0 g). The 50% withdrawal threshold was determined using Dixon’s up–down method, modified by Chaplan *et al.*[79]. First, an intermediate von Frey hair (0.16 g) was applied perpendicular to the hind-paw surface with sufficient force to cause a slight bending of the filament. In case of a positive response (rapid withdrawal of the paw within 3 s), the next smaller filament was tested. In case of a negative response, the next larger filament was tested.

### Chemicals

Fura-2 AM, Pluronic F-127 and Sulforhodamine 101 (SR-101) were purchased from Invitrogen (Carlsbad, CA). Glutamate was purchased from Sigma (St. Louis, MO). CNQX, GYKI 52466, AP-5, UBP-301, UBP-310 and AIDA were from Tocris Bioscience (Ellisville, MO).

### Statistical Analysis

Outcomes were described for the experimental conditions using means ± SEM. For each glutamate-evoked response, a sample of cells was obtained from each slice and was averaged as replicates. Data were analyzed using GraphPad Prism 5 software (GraphPad Software, San Diego, CA). One-way ANOVA (with repeated measures where applicable) was used and Dunnett’s multiple comparison test was run (unless indicated otherwise) to determine significance compared to vehicle. For SNI *vs.* sham experiments statistical tests were performed using SAS v9.3 (Cary, NC). Slices were treated with different concentrations. For each concentration and slice, the cells were treated as replicates and values for the cells were averaged. A repeated measures ANOVA was implemented with experimental condition (SNI and sham) as the between factor and concentration (0.03, 0.1, 0.3 and 1 mM) as the within factor. Pairwise comparisons were implemented using Bonferonni adjustments.

## Competing interests

The authors declare that they have no competing interests.

## Authors’ contributions

SD participated in the conception and design of the study, carried out experiments, collected and analyzed data, and drafted and critically revised the manuscript. CB collected and analyzed data for the electrophysiology experiments. BNS participated in the conception and design of the electrophysiology experiments and critically revised the manuscript. BKT oversaw the conception, design, and completion of the study, and critically revised the manuscript. All authors read and approved the final manuscript.
